# Salivary Markers as Potential Stress Descriptors for Pediatric Dental Patients: A Literature Review

**DOI:** 10.3390/children12040500

**Published:** 2025-04-14

**Authors:** Shelby Main, Marcela R. Carrilho, Anna Alessandri-Bonetti, Caroline Sawicki, Jahnavi Rao, Sheila Hall, Linda Sangalli

**Affiliations:** 1 College of Biomedical Science, Midwestern University, Downers Grove, IL 60515, USA; shelby.main@midwestern.edu; 2 College of Dental Medicine, Midwestern University, Downers Grove, IL 60515, USA; mcarri@midwestern.edu (M.R.C.); jrao@midwestern.edu (J.R.); shall1@midwestern.edu (S.H.); 3 Institute of Dental Clinic, A. Gemelli University Policlinic IRCCS, Catholic University of Sacred Heart, 00168 Rome, Italy; anna.alessandribonetti01@icatt.it; 4 Department of Pediatric Dentistry and Dental Public Health, Adams School of Dentistry, University of North Carolina, Chapel Hill, NC 27599, USA; caroline_sawicki@unc.edu

**Keywords:** salivary alpha-amylase, dental stress analyses, biomarkers, dental anxiety, dental fear, pediatric dentistry, test anxiety questionnaires

## Abstract

Dental fear and anxiety are frequently identified as major contributing factors to non-compliance, uncooperativeness, and difficulties during dental procedures in pediatric patients. These issues can lead to avoidance of dental treatment, resulting in long-term negative consequences for oral health and overall well-being. The assessment and quantification of psychological functioning (i.e., dental fear, anxiety, and self-perceived stress) has traditionally relied on self-reported questionnaires validated for the pediatric population. While this approach is cost-effective and non-invasive, it relies on subjective self-reported data, oftentimes influenced by parental or guardian interaction, especially in young children. Salivary diagnostics has recently emerged as an objective method for the procurement of biological molecules that serve as biomarkers for a variety of oral and systemic conditions. This literature review aims to comprehensively summarize the available literature on the correlation between psychological and salivary physiological measurements assessing dental fear, dental anxiety, and self-perceived stress in pediatric dental patients, highlighting the advantages and disadvantages of each method of assessment. Four databases (PubMed^®^, PsycInfo, Dentistry & Oral Sciences Source, and Web of Science) were searched for published articles, in the English language, assessing the correlation between psychological and physiological distress in children undergoing dental procedures. Studies on pediatric patients reveal positive correlations between salivary cortisol and dental fear, stress, and anxiety, especially in returning patients. Conversely, findings on salivary alpha-amylase and secretory immunoglobulin A were inconsistent, with some studies suggesting correlations with dental fear and prior dental experiences.

## 1. Introduction

Tooth decay continues to be the most common disease amongst children in the U.S., and access to dental care is often out of reach for many, contributing to a growing global health problem. Numerous barriers have been identified as contributors to oral health disparities. According to Reich et al., they can be broadly categorized into structural, attitudinal, knowledge-based, and behavioral factors [1]. These barriers often derive from children being raised in situations of poverty, low parental educational attire, family demographics, lack of access to properly educated dental providers, and geographic disparities in care, among others [1]. Such inequalities particularly affect underserved populations, further intensifying the oral health disparity.

Significant behavioral factors contributing to poor oral health outcomes in children include dental anxiety and dental fear. Dental anxiety is a state of apprehension in anticipation of a negative dental experience, while dental fear is an in-the-moment response to perceived threatening stimuli and contributes significantly to the development of dental anxiety [2]. These domains are known to lead to lack of compliance, avoidance of dental treatment, and challenges during dental procedures [3]. Their prevalence has been estimated to vary considerably from 3% to 43%, according to specific populations, assessment tools, and methodological differences [4]. In underserved pediatric populations, estimates of dental anxiety are even greater [4]. Moreover, differences across age groups and sexes have been reported. For example, a growing body of literature has suggested that dental fear and dental anxiety are more frequent among young girls and tend to decrease as age progresses [5,6,7,8].

Roughly 10–20% of adults experience dental anxiety [9], which has often developed during childhood [10], due to traumatic dental experiences or indirect observation of the parents [11,12]. Notably, patients who report dental fear are suggested to be three times more likely to miss their dental appointment compared to patients who do not report fear [13]. Moreover, performing dental procedures becomes more challenging when a child exhibits dental fear and anxiety, as these are often accompanied by maladaptive and avoidance behaviors [14]. A causal model was described long ago by Berggren, whereby fear and anxiety may lead to avoidance, which in turn exacerbates oral health issues [15]. This deterioration may intensify feelings of inferiority and shame, which in turn perpetuate a vicious cycle of fear and anxiety [3]. As a result, avoidance of dental procedures due to fear and anxiety lead to aggravation of the dental pathology and deterioration of patients’ overall oral health.

Dental fear and anxiety not only impair treatment adherence but also exacerbate the oral health disparities already prevalent among racial and ethnic minority groups. Individuals are impacted daily from neglected oral care, and these impacts have resulted in decreased quality of life from missed school days and decreased work productivity [16,17]. Moreover, low-income and minority children have fewer preventive measures, such as sealants, and attend dental appointments less frequently [18], which additionally could further deteriorate their self-perceived quality of life. Despite the significant impact of dental anxiety and dental fear, few clinicians routinely address these issues during pediatric care, underscoring the need for more comprehensive approaches in dental practice.

Psychological distress related to dental procedures is usually reported as self-perceived stress, which refers to the feelings or thoughts an individual has regarding how much stress they are experiencing during a specific period [19]. The relationship between perceived stress and dental anxiety has been demonstrated in the past, and has indicated that as levels of perceived stress increase, so does dental anxiety [20]. In children, dental fear and dental anxiety are commonly measured through questionnaires adapted and validated in this specific population, including the Children’s Fear Survey Schedule—Dental Subscale (CFSS-DS) [21] and the Modified Child Dental Anxiety Scale (MCDAS) [22]. To measure self-perceived stress in children, the Perceived Stress Scale for Children (PSS-C) is one of the most common assessment tools [23]. Despite being validated, these questionnaires gather only self-reported outcomes and thus rely on subjective measurements that can be greatly influenced by multiple sorts of bias (i.e., response, acquiescence, cognitive bias). Moreover, some of these scales (e.g., PSS-C) only assess psychological distress in the past (i.e., in the past week), thus potentially incurring recall bias and not necessarily correlating to the present situation.

The development of fear, anxiety, and stress in children results as a physiological activation of the sympathetic nervous system, specifically, the sympathetic adrenal medullary system and the hypothalamic–pituitary–adrenal (HPA) axis [24]. Cortisol and alpha-amylase are considered reliable stress-related biomarkers, reflecting sympathetic activation and HPA functioning [25,26]. Because these molecules are found in physiological levels in saliva, they can be promptly quantified through salivary collection. Due to its non-invasive nature, saliva collection is not expected to increase stress in the patient. [26]. In addition, saliva collection provides means for an easy, hygienic, and cost-effective diagnostic method [27]. Accordingly, the quantification of stress-related biomarkers can be proposed in substitution of or in combination with self-reported psychological outcomes. Ideally, from a diagnostic standpoint, it would be advantageous if psychological and physiological stress-related outcomes were concordant and positively associated.

Therefore, the current study aimed to comprehensively review available psychological and salivary physiological measurements assessing dental fear, dental anxiety, and stress in pediatric patients undergoing dental procedures, highlighting the advantages and disadvantages of each assessment. A secondary aim was to summarize the existing literature on the relationship between psychological and physiological assessment of distress in pediatric patients undergoing dental treatment.

Understanding the factors contributing to dental anxiety and stress in this population is crucial to helping develop effective interventions and improving overall oral health during childhood, with important long-term oral health consequences in later years.

## 2. Materials and Methods

A comprehensive literature review was conducted from April to August 2024 on 4 databases (PubMed^®^ via MEDLINE, PsycInfo, Dentistry & Oral Sciences Source, and Web of Science) for articles published in English language on psychological and physiological distress in the pediatric population (<18 years old) before dental procedures. Only articles that assessed both physiological and psychological measurements in the pediatric population without any further intervention and examined their correlation were included in this literature review. Studies conducted on adults, not quantifying nor assessing the relationship between psychological (stress, anxiety, fear) and acute physiological stress as measured by saliva samples, not related to any dental procedures, nor in English language were excluded. We also excluded case reports, systematic reviews, meta-analyses, and literature reviews. The search strategy utilized a combination of MeSH terms and keywords, including “dental fear” OR “dental anxiety” OR “stress” AND “pediatric patients” OR “pediatric population” OR “children” OR “adolescents” AND “dental procedure” OR “dental care” AND “salivary biomarkers” OR “salivary cortisol” OR “salivary alpha-amylase”. Boolean operators (“AND”, “OR”) were utilized to refine the search. Reference list of included articles, existing reviews, systematic reviews, and meta-analyses were manually screened to ensure comprehensiveness. Many of the available studies investigated other factors, such as the type of dental procedure, salivary flow volume, the presence and number of caries lesions. However, these factors are outside the scope of this review.

## 3. Results

**Figure 1:**
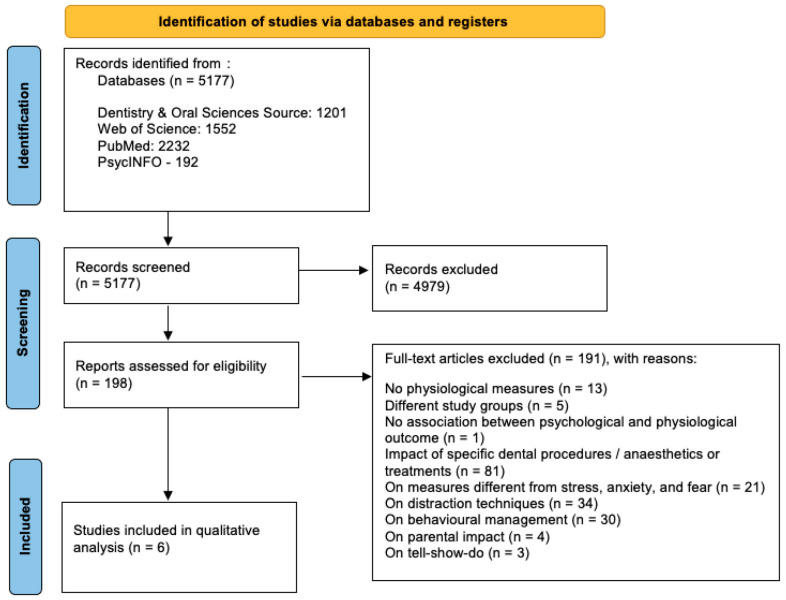
Study selection process.

The study selection process is summarized in Figure 1.

### 3.1. Psychological Measures of Dental Fear, Self-Perceived Stress, and Dental Anxiety

**Figure 2:**
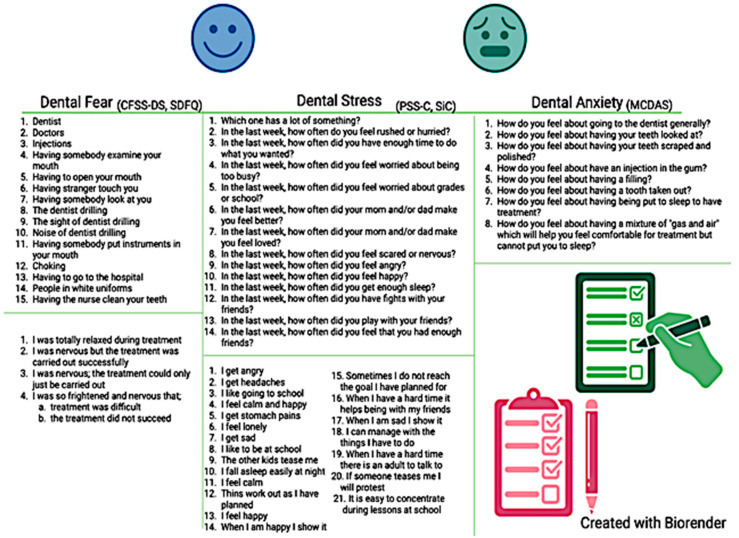
Common questionnaires assessing psychological distress used in clinical pediatric research.

Questionnaires specifically validated for the pediatric population have been used by some clinicians to measure psychological functioning at baseline so that they can anticipate and better manage potential behaviors driven by psychological distress in their young patients [28]. In this instance, the dental care provider is required to invest more time explaining the purpose and the steps of each dental procedure and consequently address concerns of a fearful or anxious patient [14]. In the presence of moderate-to-severe psychological distress, the clinician could also personalize the procedure and adopt certain considerations to decrease dental anxiety. These strategies include the use of a calming voice, “tell–show–do” procedures, or distracting techniques (including music or movies during the dental treatment), among others [29]. Repeating the administration of the questionnaire at later appointments allows for monitoring longitudinal changes of psychological distress over time [30]. Some of the most utilized questionnaires to assess psychological functioning in children are presented in Figure 2. Three of the included studies utilized the scales illustrated in Figure 2 to assess self-perceived stress and dental anxiety; two studies utilized similar scales assessing dental anxiety, while one study used ad hoc questions.

#### 3.1.1. Assessment of Dental Fear

The most commonly used instrument to quantify dental fear in pediatric patients is the Children’s Fear Survey Schedule—Dental Subscale (CFSS-DS) [21]. The CFSS-DS consists of 15 items, each representing hypothetical scenarios occurring during a dental procedure, with Likert-like scale answers ranging from 1 = “Not afraid at all” to 5 = “Very much afraid” (Figure 2). The final score ranges between 15 and 75, with values ≥ 38 being consistent with dental fear [22]. Its use has been validated in children ages 7–12 [22]. Contradictory results emerge from the existing literature on whether dental fear may differ across age groups, with a study revealing no statistically significant difference between mean dental fear score across various age groups within adolescents [31]. Validity, time completion, and ease of use of the CFSS-DS make this questionnaire one of the most reliable. In relation to sex, one study showed significantly higher dental fear in girls compared to boys [32,33]. Another available instrument to measure dental fear is the Short Dental Fear Question (SDFQ) [34], which has been tested on teenagers, and consists of queries investigating how the dental procedure was completed [34]. The SDFQ is quick, easy to answer, and requires minimal advanced knowledge for interpretation [35].

#### 3.1.2. Assessment of Self-Perceived Stress

Self-perceived stress has been commonly measured in the pediatric literature through several validated questionnaires. The most common is the Perceived Stress Scale for Children (PSS-C), a 13-item questionnaire with option choices ranging from 0 = “Never” to 3 = “A lot” (Figure 2). The final score ranges from 0 to 39, with higher values indicating greater symptomatology [23]. The items present different scenarios that could potentially evoke a state of mental stress. Specifically, items 2, 4, 5, 8, and 12 examine stressor sensitivity related to everyday scenarios; items 8, 9, 10, and 12 assess emotional state; items 6 and 7 investigate security; and items 2, 3, and 4 are considered time pressure questions. This questionnaire has been validated among various populations including dentists, healthcare students, and pediatric patients [23,36,37]. Another questionnaire developed and validated to assess self-reported stress is the Perceived Stress Scale for Kids (PeSSKi) [38], a 10-item assessment investigating feelings and thoughts a child remembers within the past month. Similarly, the 21-item Stress in Children (SiC) presents several factors regarding emotions and physical health (e.g., headaches, stomach pain) [39]. Another validated questionnaire indirectly assessing stress and mental well-being is the Child Perception Questionnaire (CPQ), which intrinsically examines the children’s quality of life and mental well-being [40].

#### 3.1.3. Assessment of Dental Anxiety

Several measures have been validated in clinical research to assess dental anxiety. One of the most utilized assessment tools is the five-item Dental Anxiety Scale (DAS), which has been modified over time to the Modified Child Dental Anxiety Scale (MCDAS, Figure 2) [41]. Found to be most valid in children ages 8–12, this questionnaire consists of eight items, characterized by five-point Likert scale answers and anchors ranging from 1 = “Relaxed/not worried” to 5 = “Very worried” [41]. A final score is obtained by summing all item scores, and it ranges from 8 to 40. A cut-off of 19 has been established to detect the presence of anxiety symptomatology, with categories of 8–10 = no anxiety, 19–30 = presence of anxiety, and 31–40 = severe anxiety. Dental anxiety has been positively correlated with dental fear in a study conducted on children 8–12, regardless of the type of questionnaire utilized [41].

#### 3.1.4. Advantages and Disadvantages of Psychological Assessment in Children

The use of validated questionnaires to assess and quantify psychological distress in children has been shown to provide additional means to assess children’s cognitive perspective with good reliability [42]. For instance, they allow for the assessment of the presence and degree of severity of psychological distress in a cost-effective manner. However, while useful, validated questionnaires are insufficient for establishing a conclusive diagnosis. A proper diagnosis requires meeting criteria as established by the Diagnostic and Statistical Manual of Mental Disorders (DSM-5) or the International Classification of Disease (ICD-10) [43], which is normally provided by a licensed psychologist, social worker, or psychiatrist. Moreover, these questionnaires rely solely on subjective self-reported data. Another potential issue is the reliability of the answers of the pediatric patients, who could potentially provide false responses to avoid showing signs of fear or based on social desirability. Given these limitations, it is likely that using objective measures of psychological distress in conjunction with self-reported questionnaires could greatly enhance the identification, quantification, and monitoring of symptoms in a reliable manner, to ultimately improve treatment care.

### 3.2. Psychological Measures Through Salivary Collection

#### 3.2.1. Salivary Diagnostics

**Figure 3:**
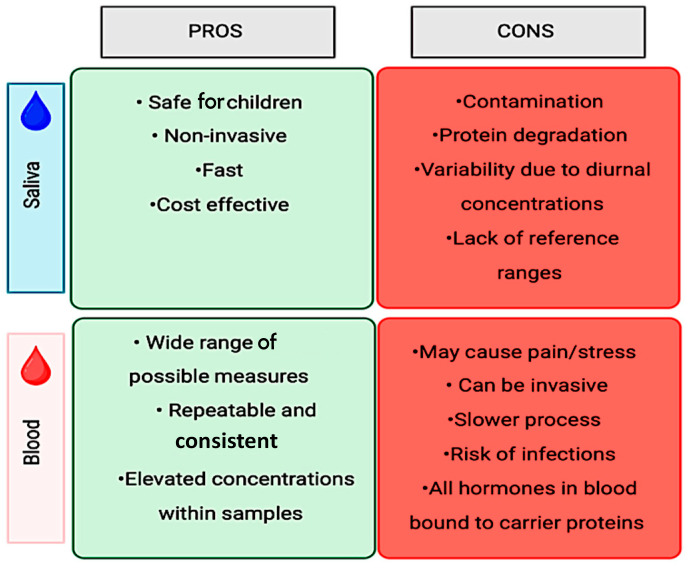
Salivary vs. blood sample collection.

The most common methods proposed to objectively measure stress involve the collection of stress markers from bodily fluids, such as blood and saliva. Traditionally, clinical and experimental studies have utilized blood tests as a standard method of collection to identify and measure markers, make diagnoses, and monitor the progression of the condition. However, blood collection is not only an invasive method, but, especially in young patients, it is perceived as causing more discomfort and pain [44], in addition to being associated with a greater risk of possible infections depending on the volume extracted [44] and being potentially stressful [45]. Thus, the use of saliva as a diagnostic method to quantify molecules associated with stress offers a substantial advantage for the advancement of this field of research. Salivary diagnostics has proven to be a valid alternative for diagnosing and monitoring disease progression [46]. Among other benefits, salivary diagnostics is considered to be a minimally invasive, cost-effective, and less uncomfortable approach compared to blood diagnostics, especially for the pediatric population. The advantages and disadvantages of both quantification methods are summarized in Figure 3.

Salivary diagnostics consists of the collection of either the whole or salivary-gland-specific saliva and the quantification of the molecules within this biological fluid. The salivary glands are divided between the major and minor glands, which all perform the function of producing and secreting saliva into the oral cavity [47]. The three pairs of major salivary glands, from largest to smallest, are the parotid, submandibular, and sublingual. They are responsible for secreting the greatest volume of salivary fluid (~90%), which differs substantially between them in terms of composition and fluidity [47]. The different types of acinar cells in each major salivary gland result in different types of saliva production. While the parotid gland has serous acini and produces a watery serous saliva, the submandibular and sublingual glands are mixed glands, containing mucous and serous acini in different proportions. Consequently, since most of the submandibular acinar cells are serous, they produce more fluid saliva in contrast to the sublingual glands, which are composed primarily of mucous acinar cells [47,48,49]. Due to their proximity to blood vessels, salivary glands are a rich source of metabolite exchange between the oral cavity and the circulatory system [49]. In fact, many proteins found in human serum can also be detected in saliva, supporting its use as a proxy for the measurement of circulating biomarkers related to diseases [50].

Under normal physiological conditions, children produce on average 1.5 L of saliva per day [51]. The saliva can be secreted under unstimulated and stimulated conditions, where unstimulated salivary flow will result from salivary gland secretions in a resting state (~66% of the whole saliva) [51], while stimulated saliva (the remaining ~33%) is obtained by activating the entire multitude of salivary glands, major and minor, through the use of chemical substances or mechanical stimuli (e.g., chewing). Normal levels of stimulated and unstimulated saliva are shown in Table 1. Saliva production is susceptible to overproduction, a condition known as sialorrhea particularly seen in children [52]. The largest heterogeneity among saliva in individuals is the result of the sympathetic and parasympathetic effects of the salivary gland from diseases, medications, and pharmacological interventions, which can significantly influence the secretion of saliva volume and additionally lead to alterations in fluid composition [49,53].

**Table 1:** Reference ranges of salivary flow/salivary concentration in children.

Measures	Unstimulated Whole Saliva	Stimulated Whole Saliva	Stimulated Parotid Saliva
Normal Reference Range in Saliva	0.08–3.30 mL/min [54]	0.25–5.58 mL/min [54]	0.04–0.69 mL/min [54]
Reference Range under Psychological/ Physiological Stress	INCREASE [55] Range of values differs upon maximum volume of saliva in individuals.	INCREASE [56] Range of values differs upon maximum volume of saliva in individuals.	DECREASE [51] Range of value differs upon maximum volume of saliva in individuals.

**Figure 4:**
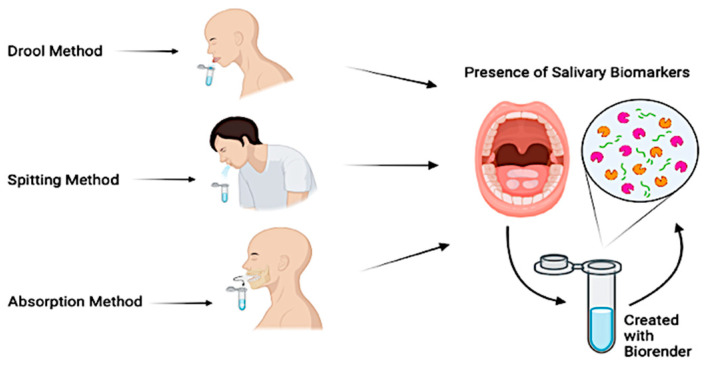
Saliva collection techniques.

The most common saliva collection techniques consist of drooling, absorption, or spitting (Figure 4).

The *drooling technique*, while not commonly used in children, is considered the gold standard for unstimulated saliva collection [57]. In an upright position with the head tilted forward, the patient is instructed to collect the saliva and to empty it into a collection container. Given its ability to provide a high salivary flow rate with minimum effort required by the patient, it has consistently been selected as the primary collection method across several studies. The *absorption technique* collects both stimulated and unstimulated saliva and is often considered the method of choice within pediatric patients, thanks to the ease of collection and minimal time required. This technique consists of providing the patient with a dedicated tool that is often chewed on for a period from 60 s to 2 min, to allow for saliva absorption [58]. The *spitting technique* can be used both in children and adults and collects both stimulated and unstimulated saliva. This method consists of instructing the patient to spit every 30 s directly into a collection device below the chin, for a 2-to-5 min collection period [59]. Multiple studies have also proposed the combination of multiple collection techniques to either compare methods or assess the efficacy of stimulated vs. unstimulated saliva [60,61]. Each of these techniques offers advantages and disadvantages, while collecting different types of saliva. The researcher is ultimately expected to determine which technique is the most suitable for their patients and compare the results to established reference values [62,63] (Table 1).

#### 3.2.2. Salivary Molecules

With the advance of salivary collection methods and analyses, clinicians and researchers can measure saliva volume and its biochemical composition [64]. This is particularly useful for those molecules that can be quantified within human body tissues and fluids in short response times (e.g., cortisol, alpha-amylase, immunoglobulins, C-reactive protein, and cytokines), all being produced across different physiological stages within the body. Notably, the concentration and volume of some of these molecules have been suggested to differ according to sex, age group, systemic medications and conditions, and diet [64]. Table 2 shows the normal values of the stress-related salivary molecules for pediatric populations and level changes according to the collection method used. The most common molecules quantifiable through salivary samples and related to stress in the existing literature are cortisol and alpha-amylase, which have been frequently assessed in children [59,65,66,67,68,69].

Salivary cortisol: Salivary cortisol is a well-known and reliable molecule released as a result of hypothalamic activation [70]. This hormone is produced within the adrenal cortex and can be measured both within the plasma and saliva. Cortisol is responsible for many human functions, including restoring the body’s homeostasis and regulating blood pressure and circadian rhythm, along with many others [71]. The highest levels of cortisol are present early in the morning, which then progressively decrease throughout the day. As cortisol levels are influenced by the circadian rhythm cycle, its collection needs to be performed at a consistent time during the day, to allow for within- and between-subject comparison [71]. Sex is also known to influence reference ranges of cortisol, although the literature has shown contradictory findings. For example, while older studies observed a 20% higher cortisol levels in girls between the ages of 8–16 years compared to age-matched boys [72], others have failed to reveal any significant differences across sexes [73]. Other studies showed a correlation between cortisol and social behaviors in male adolescents, but not in girls [74].

Alpha-amylase: Alpha-amylase (sAA) is a protein found in saliva whose levels are oppositely correlated to salivary cortisol [75]. The normal expected values of salivary alpha-amylase are suggested to progressively increase throughout the day [26]. Its function consists of hydrolysis and carbohydrate breakdown, secondary to caloric intake [25]. It is mainly produced and excreted by the pancreas and salivary glands [26]. In recent years, salivary alpha-amylase has been extensively studied and has been suggested as an effective biomarker of dental fear [76] and dental anxiety [77]; persistently inconclusive findings relate sAA with pain intensity [68,78,79].

Immunoglobulins: Among the primary immunoglobulins (IgA, IgG, IgM) found in saliva, IgA is the class with the highest concentration in saliva (sIgA). According to Alaki et al., sIgA can be used as a biomarker of stress in individuals [69]. Nevertheless, other studies showed that perceived stress and anxiety were correlated with low salivary IgA, which further decreased during the body’s response to stress [80].

Less common stress-related salivary molecules: There are few other stress-related salivary molecules that have been less commonly studied in children. For example, C-reactive protein (CRP) is an acute non-specific immune inflammatory biomarker that is commonly measured in saliva. The total protein concentration is affected by both CRP and immunoglobulin concentrations, specifically IgA [69]. Levels of CRP have been correlated with cardiovascular diseases; thus, quantification of CRP has also been utilized for diagnostic reasons and follow-ups [81]. Other alternative molecules are catecholamines, such as norepinephrine (NE) and epinephrine. Salivary NE levels were correlated with stress due to the sympathetic response caused by restorative dental procedures with local anesthesia [82]. Interestingly, NE returned to baseline levels after treatment. Conversely, salivary epinephrine levels were not correlated with dental anxiety [82].

**Table 2:** Reference ranges of concentrations of salivary molecules in children.

Salivary Molecule	Salivary Cortisol	Salivary Alpha-Amylase (sAA)	Salivary Immunoglobulin A (sIgA)	C-Reactive Protein (CRP)	Cytokines
Normal Reference Range in Saliva	AM: 3–19 mcg/dL PM: 1–11 mcg/dL	<10 mg/L [83]	1–220 mg/dL [84]	0.1 mg/L–6 mg/L (levels should not exceed 10 mg/L) [85]	IL-2: 11.91 ± 1.70 (pg/mL) IL-1β: 64.5 ± 89.6 (pg/mL) IL-6: 5.2 ± 2.8 (pg/mL) IL-8: 210.096 ± 142.302 (pg/mL) IL-10: 12.02 ± 7.23 (pg/mL) [86]
Reference Range under Psychological/ Physiological Stress	159.83 ± 460.36 [87]	>10 mg/L [83]	Increase from existing studies. Pre-treatment = 1116.1–1121.7 µg/mL; Post treatment = 1200.6–1210.8 µg/mL [88]	Increase in the presence of psychological stressor [89]	Increase in the presence of psychological stressor [89]

#### 3.2.3. Advantages and Disadvantages of Salivary Stress-Related Physiological Measures

Salivary diagnostics and the quantification of pathogens and protein content can be utilized for the diagnosis and monitoring of the onset and progression of certain conditions [90]. Nevertheless, these findings should be used in conjunction with clinical considerations to achieve the most appropriate diagnosis.

While salivary diagnostics is a cost-effective, non-invasive procedure, especially for pediatric patients, the collection technique and timing, the protocol in place for storage, and the participant inclusion should be standardized and carefully planned, as they may contribute to errors and variations across study participants. For example, inconsistencies in results can arise from several factors, including the timing of salivary collection, as some of the salivary molecules fluctuate throughout the day due to circadian rhythms. Salivary samples need to be stored in ice soon after their collection, as the improper handling of samples can lead to variations in the salivary proteome [91]. Failure to use the recommended storing of samples based on the brand used can lead to an increased rate of degradation and bacterial growth [92].

### 3.3. Relationship Between Psychological and Physiological Distress in Pediatric Patients

While many studies in adults suggest that changes in the levels of salivary molecules may be related to stress and anxiety related to dental procedures [65,93,94,95], limited research has explored this correlation in children using validated questionnaires. The use of validated questionnaires is necessary to quantify measures of psychological distress with established validity and reliability.

Table 3 summarizes the correlation, or the lack thereof, between psychological and physiological stress-related outcomes in the pediatric literature.

Most of the literature exploring the relationship between psychological and physiological distress in children has been published in the last decade [58,69,88,96,97,98]. All of these studies consistently used salivary cortisol as the primary physiological measure, to such an extent that cortisol can indeed be considered a reliable salivary biomarker. Salivary cortisol levels were found to be significantly and positively correlated with dental fear [96], stress [69], and anxiety symptoms [88,98]. Three studies found that the concentration of salivary cortisol was significantly greater in returning patients compared to new patients [69,88,97]. For example, Aksoy et al. investigated the salivary cortisol and anxiety levels in children starting an orthodontic treatment with multibracket fixed appliances. The highest level of cortisol was observed at the end of the second appointment compared to the first appointment, with values of 0.57 ± 0.07 and 0.59 ± 0.03 (according to the type of archwire utilized) vs. 0.39 ± 0.03 and 0.41 ± 0.04 at the beginning of the first appointment [97]. Similarly, the participants exhibited the highest levels of anxiety at the end of the second appointment, reflecting cortisol levels [97]. In another study, Dhinsa et al. found that cortisol levels measured at the end of the second appointment (0.32 ± 0.06) were significantly greater compared to those measured at the end of the first appointment (0.13 ± 0.02, *p* < 0.0001) [88]. Likewise, another study confirmed that, compared to new patients, returning patients exhibited 0.20% greater levels of salivary cortisol [69]. This potential difference between new and returning patients may not have been observed by those studies characterized by a cross-sectional design, thus not allowing for a longitudinal evaluation over different timepoints (e.g., [58,98]). Conflicting findings were found on the influence of sex on salivary cortisol levels, with Alaki et al. observing higher levels in male patients [69], while other studies supporting no significant difference between female and male patients [97,98]. Interestingly, Alaki et al. observed that the children whose saliva was collected by a male provider presented with greater stress levels (*p* = 0.05) than when a female provider conducted the procedure [69]. This factor is often normalized across the studies by using one experimenter, which certainly reduces confounding factors related to the provider, but could overlook some other important considerations such as the influence of the experimenter’s sex on the results. Within the adult population, increased levels of salivary cortisol have been associated with greater self-perceived stress and periodontitis severity. For example, a case–control study conducted on 120 adults revealed that those with periodontitis exhibited on average >50% higher salivary cortisol and stress levels compared to healthy controls (*p* < 0.001) [99], results which were confirmed by a meta-analysis of observational studies [100]. Conversely, Sadi et al. failed to reveal significant correlations between dental anxiety and salivary biomarkers, including salivary cortisol and sAA [77].

Four of the six studies also included sAA, with contradictory findings on its reliability as a stress- or fear-related salivary biomarker, and with differences across collection times. For example, overall sAA levels were found to be significantly and positively correlated with dental fear, especially in children undergoing dental procedures with local anesthetics [58]. Differences were found between the first and second appointments, in that sAA levels significantly correlated with dental fear at pre-intervention during the first appointment, while they correlated with dental fear at the second appointment only after the intervention [88]. Similar conclusions have been suggested by Alaki et al., who confirmed significantly higher levels of sAA in returning patients compared to new patients [69]. Moreover, in new patients, sAA was greater when measured in the dental chair (i.e., immediately prior to starting the intervention) compared to when it was measured in the waiting room [69]. This fluctuation may be attributed to expectations and prior dental experience. Levels of sAA were also found to positively correlate with dental anxiety [96]. In the adult literature, a study claimed that sAA levels were positively correlated with dental anxiety before the dental procedure, which decreased at post-treatment [101]. Conversely, some others failed to reveal any correlation between sAA levels and dental anxiety, although dental anxiety was positively correlated with intraoperative pain [102] and a history of traumatic events [77].

The overall literature in adults does not seem to attribute any influence of dental fear, dental anxiety, and stress on the change of sIgA. However, in studies conducted in the pediatric population, sIgA seemed to follow a trend similar to sAA [88]. For example, sIgA levels were observed to be higher in returning patients compared to new patients [69]. Moreover, sIgA was also proposed to be correlated with dental anxiety at pre-intervention during the first appointment, and at post-intervention during the second appointment [88].

Altogether, this suggests that past dental experience may be a contributing factor. Approximately 50% of the studies suggested that results fluctuate depending on the sex of the patients [58,69,88,96,97,98].

## 4. Discussion

The current study aimed to summarize psychological assessment tools available in the pediatric literature to evaluate the presence and intensity of perceived stress, anxiety, and fear related to dental procedures and to correlate these measures to acute physiological measures of stress. Despite contradictory evidence derived from studies conducted on adults, the available literature in pediatric patients showed a substantial agreement between measures of psychological and acute physiological distress.

The pediatric population is largely affected by fear and anxiety while facing dental procedures. These conditions play a major role in influencing poor oral health outcomes, as they lead to non-compliance, avoidance of dental care, and difficulties during procedures [103]. Moreover, social and ethnic minority groups, particularly low-income populations, often face greater barriers to preventive dental care, such as fewer visits and more missed appointments [104]. This impacts both overall health and economic productivity, with studies showing that poor oral health is correlated with lost workdays.

As seen throughout this review of the literature, there is an increasing interest in the scientific community in identifying useful stress-related molecules that could be utilized as stress biomarkers to assess dental anxiety, dental fear, and stress in pediatric patients. In this review, we have summarized existing physiological and psychological instrument tools available in the literature. Further research, using combinations of methods, is expected to continue providing evidence on the correlation of these assessment tools.

Overall, our findings suggested a relative concordance between psychological measures assessed through validated questionnaires and levels of acute physiological stress-related molecules. There was a consistent positive relationship between dental anxiety and dental fear and levels of salivary cortisol [58,88,96,98], which confirms salivary cortisol as a valuable biomarker of psychological distress in pediatric dental patients. Interestingly, differences were seen between new patients and returning patients. For example, the fact that returning patients may experience higher levels of cortisol, sIgA, and sAA (and, thus, of fear and anxiety) suggests that the same approaches adopted by the pediatric dentist during the first visit to create a comfortable and friendly environment may need to be replicated for returning patients. SAA was also consistently related to increased psychological distress, adding this as a reliable physiological marker of stress.

The findings have significant implications for pediatric dentistry, by highlighting the importance of managing dental anxiety and fear to improve overall patient outcomes and by contributing to the development and exploration of non-invasive diagnostic tools and targeted interventions to enhance patient comfort and cooperation during dental treatments. There are many preventive measures that could be included in all appointments, known to lessen children’s fear during their dental visits. These include scheduling dental appointments at a time that best accommodates the child, as well as increasing the frequency of visits for children to become more familiar with the environment [14] and enhancing the patient’s involvement and interactive approaches (i.e., tell–show–do) to increase the child’s sense of comfort [39].

Nevertheless, saliva collection and the detection of salivary molecules within a dental office would additionally require advanced technology [105] and access to laboratory spaces meeting biosafety requirements. While these analyses may be possible at university-affiliated dental clinics with interdisciplinary departments, a routine assessment of salivary biomarkers may not be a feasible option in private clinical practices. Yet, confirmation of self-reported psychological outcomes with physiological measurements could potentially help tailor the way dental procedures are completed [106], contributing to providing more comprehensive care and procedural explanation to children who present with fear. Additionally, clinicians could help parents develop routine dental habits at home based on their children’s behaviors and reactions in the office, which could likely promote positive responses of those children presenting with psychological distress [107].

Future studies should explore other physiological measurements of distress, such as heart rate variability, and investigate their potential associations with psychological outcomes. Additionally, biomarkers of pain, such as calcitonin gene-related peptide (CGRP) or substance P, could be measured to better understand the relationship between physiological stress responses and pain perception. Future research should also consider differences between returning and new patients, examining how past traumatic experiences or lack of prior dental treatment influence physiological and psychological responses to dental care.

### Limitations

Despite the authors’ efforts to thoroughly review the topic, this study should be regarded as a literature review and not a systematic review, which may limit the generalizability of its findings. Thus, potential limitations include the selection bias of the included studies, a lack of standardized methodology across sources, and the possibility of missing relevant literature.

## 5. Conclusions

Assessment of psychological distress with validated questionnaires and quantification of physiological molecule levels appears to be relatively consistent and positively correlated in the pediatric literature. Given the several contributing factors that can enhance dental fear, dental anxiety, and stress in the pediatric population, understanding the influence of such factors during dental procedures will ultimately enable the provider to enhance the quality of care and the well-being of the pediatric patients.

## Figures and Tables

**Table 3:** Correlation between psychological outcomes and acute physiological stress in the included studies.

Author (Year)	N Participants and Subgroups	Age Range (mean ± SD); % F	Physiological Molecule	Collection Points and Methods	Psychological Outcome (Measure)	Additional Measures
AlMaummar et al. (2019) [96]	N = 151: - 50 in the anxious group, - 40 in the phobic group, - 53 in the control group	6 to 9 y/o (7.4 ± 1.2); 51% F	Salivary cortisol, sAA	Passive drool method, three times (early morning, at 12 pm, and at 8 pm) at 3 months and 1 year after dental treatment	Dental fear (15-item, CFSS-DS)	Mean heart rate; behavioral management; number of cavities; plaque accumulation
Alaki et al. (2017) [69]	N = 80: - 40 new patient, - 40 returning patients	9 to 12 y/o; (8.8 ± 9.1); 56% F	Salivary cortisol, sIgA and sAA	Measured twice on the same appointment (waiting room and dental chair)	Self-perceived stress (ad hoc question)	Unstimulated salivary flow
Noorani et al. (2014) [58]	N = 77: - 25 (no local anesthetics), - 27 (use of local anesthetic), - 25 (control group)	5 to 12 y/o	sAA	Measured twice on the same appointment (before and after treatment) through absorption technique	Dental fear (15-item, CFSS-DS)	Behavioral assessment
Dhinsa et al. (2019) [88]	N = 60	6 to 12 y/o; Male: 7.4 ± 1.3; Female: 7.3 ± 1.0	Salivary cortisol, sAA, and sIgA	Unstimulated saliva measured during 1st and 2nd appointments at pre- and post-treatment (with spitting technique)	Dental anxiety (four-item, CDAS)	Pulse rate and oxygen saturation
Aksoy et al. (2019) [97]	N = 20	12.8 ± 0.7 y/o; 50% F	Salivary cortisol	Eight collections in total (before and after four orthodontic appointments)	Dental anxiety (20-item, STAIC)	None
Vlad et al. (2020) [98]	N = 389: 170 being anxious, 219 being non-anxious	6 to 9 y/o (7.6 ± 1.3); 52% F	Salivary cortisol	One collection point	Dental anxiety (13-item, ACDAS)	Salivary flow rate using mixed saliva (stimulated and unstimulated)
**Author (Year)**	**Results**		**Conclusions**			**Correlation Between Physiological and Psychological Measures**
AlMaummar et al. (2019) [96]	*sAA*: significantly higher levels present in phobic group > anxious group > controls at 3 months (*p* = 0.029) and 1 year (*p* = 0.007), with significant differences between phobic and anxious groups. *Salivary cortisol*: significantly higher levels in phobic group > anxious group > controls at 3 months (*p* = 0.000) and at 1 year (*p* = 0.000); no significant difference between phobic and anxious groups.		Phobic patients exhibited highest levels of sAA compared to anxious patients and control group over time. Phobic and anxious patients exhibited highest levels of salivary cortisol compared to the control group.			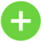
Alaki et al. (2017) [69]	*sAA*: significantly higher in returning patients compared to new patients (*p* = 0.001), especially in the dental chair (*p* = 0.019). *Salivary cortisol*: No differences between returning and new patients (*p* = 0.046); higher in new male patients compared to female patients (*p* = 0.05); returning patients had significantly higher cortisol with male provider than female (*p* = 0.02); higher in the waiting area compared to dental chair measure (*p* = 0.05). *sIgA*: higher levels in returning patients compared to new patients (*p* = 0.016); no difference between waiting room vs dental chair (*p* = 0.035).		Salivary cortisol level is increased in new patients while waiting in the waiting room; higher sAA and sIgA in returning patients; provider’s gender and location of salivary collection (waiting room vs. dental chair) influenced salivary molecule levels.			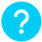
Noorani et al. (2014) [58]	sAA levels positively correlated with dental fear before (*p* < 0.001) and after the procedure in local anesthetics group (*p* < 0.001); in the group without local anesthetics, dental fear correlated with sAA levels after (*p* = 0.0005) but not before the procedure (*p* = 0.59).		Dental fear and sAA levels are positively correlated, especially when the dental procedure involves the use of local infiltration.			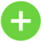
Dhinsa et al. (2019) [88]	At 1st appointment, positive correlation between dental anxiety and levels of cortisol (*p* = 0.033), sAA (*p* = 0.009), and sIgA (*p* = 0.008) at pre-intervention. Salivary cortisol and anxiety decreased at post-treatment compared to pre-treatment; sAA and sIgA levels increased at post-treatment compared to pre-treatment. At 2nd appointment, positive correlation between dental anxiety and cortisol (*p* = 0.043) at pre-intervention, but not with sAA and *sIgA*. At post-treatment, all salivary measures increased significantly and were correlated with dental anxiety.		SAA, sIgA, cortisol levels are positively correlated with dental anxiety, especially before the intervention for new patients and after the intervention for returning patients.			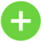
Aksoy et al. (2019) [97]	*Salivary cortisol* increased consistently with anxiety/pain levels		Anxiety levels increased over time, as well as cortisol levels and pain intensity; sex did not influence cortisol level.			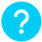
Vlad et al. (2020) [98]	*Salivary cortisol*: Female patients had greater odds of presenting with anxiety than male patients (*p* = 0.041); cortisol levels were similar between sexes (*p* = 0.02); cortisol levels were significantly higher in anxious patients compared to controls (*p* < 0.001), with moderate correlation with dental anxiety scores (*p* < 0.001)		Positive correlation between dental anxiety and salivary cortisol levels. Sex did not influence cortisol levels.			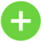

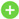
 Consistent results between physiological and psychological measures. Inconsistent results between physiological and psychological measures. 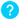 *ACDAS:* Abeer Children Dental Anxiety Scale; *CDAS*: Corah’s Dental Anxiety Scale; *CFSS-DS:* Children’s Fear Survey Schedule—Dental Subscale; F: female patients; *sAA:* salivary alpha-amylase; *sIgA:* salivary immunoglobulin A; *STAIC:* State–Trait Anxiety Inventory.

## Data Availability

No new data were created. All of the data summarized in this review are contained within the manuscript.
